# A Prospective Study on the Diagnoses for Abdominal Pain After Bariatric Surgery: The OPERATE Study

**DOI:** 10.1007/s11695-023-06756-3

**Published:** 2023-08-11

**Authors:** Nienke van Olst, Marjolein R. A. Vink, Sterre C. P. de Vet, Barbara A. Hutten, Victor E. A. Gerdes, Jeroen A. W. Tielbeek, Sjoerd C. Bruin, Stijn J. B. van Weyenberg, Donald L. van der Peet, Yair I. Z. Acherman

**Affiliations:** 1 Department of Bariatric Surgery, Spaarne Gasthuis, Spaarnepoort 1, 2134 TM Hoofddorp, The Netherlands; 2 Department of Surgery, Amsterdam UMC, De Boelelaan 1117, 1081 HV Amsterdam, The Netherlands; 3 Department of Epidemiology and Data Science, Amsterdam UMC, University of Amsterdam, Meibergdreef 9, 1105 AZ Amsterdam, The Netherlands; 4 Amsterdam Cardiovascular Sciences, Diabetes & Metabolism, Amsterdam, The Netherlands; 5 Department of Vascular Medicine Amsterdam UMC, Meibergdreef 9, 1105 AZ Amsterdam, The Netherlands; 6 Department of Radiology, Spaarne Gasthuis, Spaarnepoort 1, 2134 TM Hoofddorp, The Netherlands; 7 Department of Gastroenterology, Spaarne Gasthuis, Spaarnepoort 1, 2134 TM Hoofddorp, The Netherlands

**Keywords:** Bariatric surgery, Abdominal pain, Diagnoses, Unexplained complaints, Outpatient clinic, Emergency department, Readmissions

## Abstract

**Purpose:**

Long-term follow-up after bariatric surgery (BS) reveals high numbers of patients with abdominal pain that often remains unexplained. The aim of this prospective study was to give an overview of diagnoses for abdominal pain, percentage of unexplained complaints, number and yield of follow-up visits, and time to establish a diagnosis.

**Materials and Methods:**

Patients who visited the Spaarne Gasthuis Hospital, The Netherlands, between December 2020 and December 2021 for abdominal pain after BS, were eligible and followed throughout the entire episode of abdominal pain. Distinction was made between presumed and definitive diagnoses.

**Results:**

The study comprised 441 patients with abdominal pain; 401 (90.9%) females, 380 (87.7%) had Roux-en-Y gastric bypass, mean (SD) % total weight loss was 31.4 (10.5), and median (IQR) time after BS was 37.0 (11.0–66.0) months. Most patients had 1–5 follow-up visits. Readmissions and reoperations were present in 212 (48.1%) and 164 (37.2%) patients. At the end of the episode, 88 (20.0%) patients had a presumed diagnosis, 183 (41.5%) a definitive diagnosis, and 170 (38.5%) unexplained complaints. Most common definitive diagnoses were cholelithiasis, ulcers, internal herniations, and presumed diagnoses irritable bowel syndrome (IBS), anterior cutaneous nerve entrapment syndrome, and constipation. Median (IQR) time to presumed diagnoses, definitive diagnoses, or unexplained complaints was 16.0 (3.8–44.5), 2.0 (0.0–31.5), and 13.5 (1.0–53.8) days (*p* < 0.001). Patients with IBS more often had unexplained complaints (OR 95%CI: 4.457 [1.455–13.654], *p* = 0.009). At the end, 71 patients (16.1%) still experienced abdominal pain.

**Conclusion:**

Over a third of abdominal complaints after BS remains unexplained. Most common diagnoses were cholelithiasis, ulcers, and internal herniations.

**Graphical Abstract:**

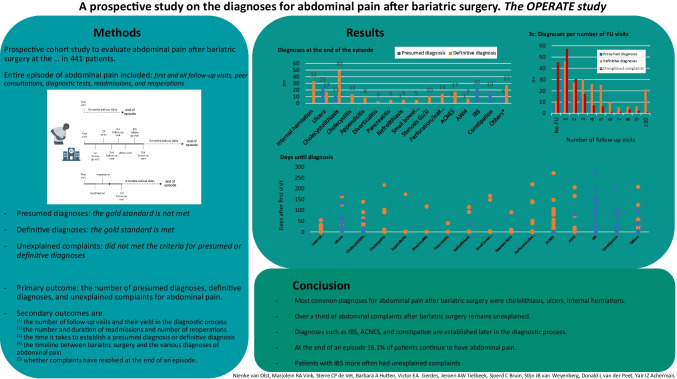

**Supplementary Information:**

The online version contains supplementary material available at 10.1007/s11695-023-06756-3.

## Introduction

The global prevalence of obesity is increasing, which impacts the number of bariatric surgeries performed [1–3]. Bariatric surgery (BS) is currently still the most effective long-term treatment for severe obesity. It has been shown to induce sustainable weight loss and improvement of obesity-related comorbidities, with low complication rates in the first 30 days after surgery [4, 5]. Long-term follow-up, on the other hand, reveals the side effects of BS such as abdominal pain, which leads to numerous hospital visits, readmissions, and reoperations [6].

The self-reported prevalence of abdominal pain after BS varies between 33.8 and 54.4% [7–9]. Abdominal pain has a negative impact on quality of life and imposes a burden on public healthcare costs [8–10]. Establishing a diagnosis for abdominal pain after BS often requires several tests, including blood tests, ultrasounds, CT-scans, gastroscopies, and diagnostic laparoscopies [11–13]. Common causes are internal herniation, an anastomotic ulcer, and gallstone disease [14–18]. However, often abdominal pain remains unexplained because a diagnosis cannot be made [12, 19, 20].

Literature on the diagnoses of abdominal pain during outpatient clinic and emergency department visits among patients after BS is limited. Crucially, follow-up of the abdominal pain after first visits is lacking, as is insight into the magnitude of the group with unexplained complaints at the end of the follow-up. The aim of this study is to provide the first overview of the diagnoses of abdominal pain in patients after BS and the percentage of unexplained complaints at initial presentation and after follow-up. In addition, number and yield of follow-up visits and the time between first visit and the diagnosis are examined. Finally, it is examined whether the complaints were resolved or persisted at the end of follow-up.

## Methods

### Study Design and Setting

**Fig. 1:**
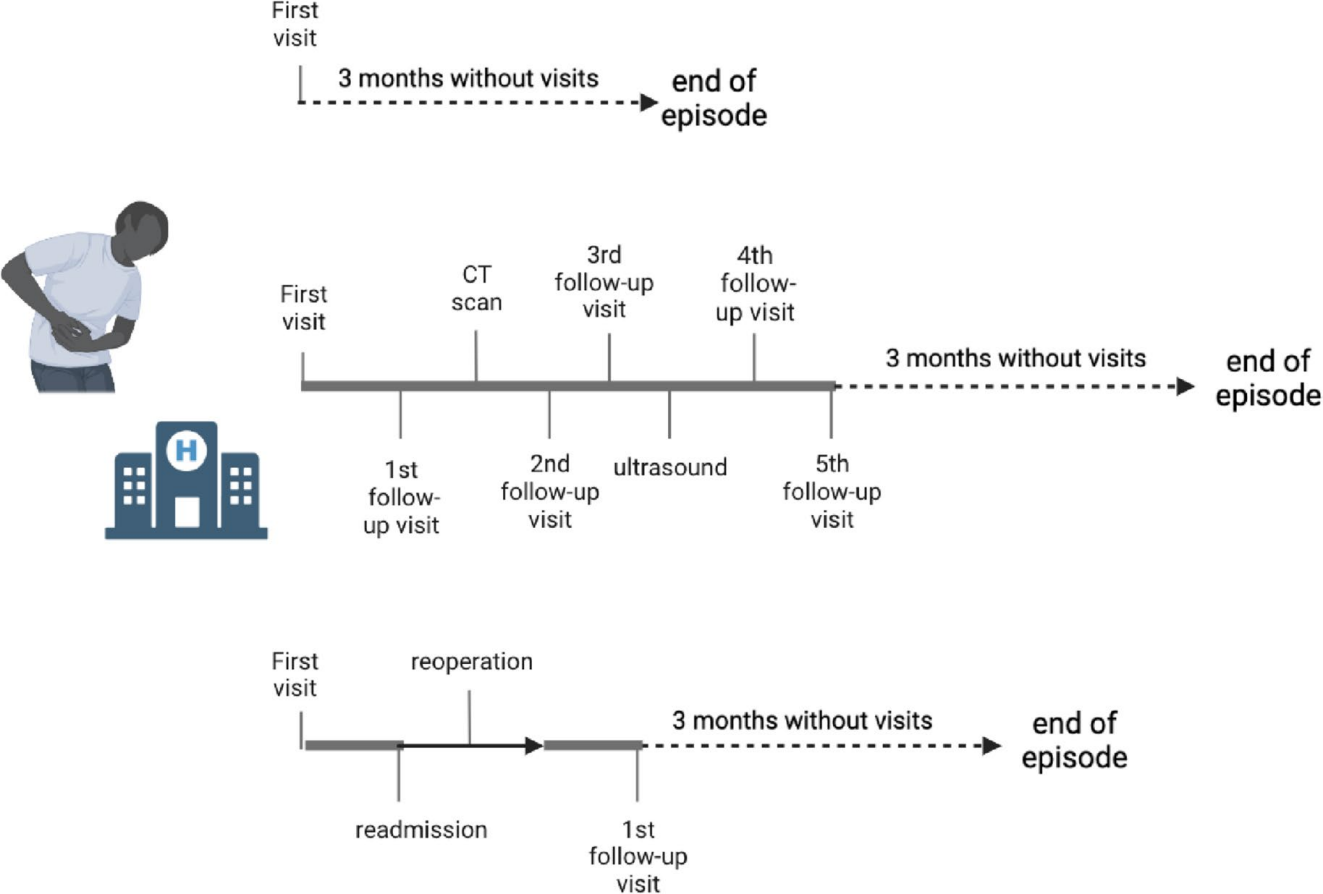
Examples of episodes

The OPERATE (abdOminal Pain aftER bAriaTric surgEry) study is a prospective cohort study to evaluate multiple aspects of abdominal pain in patients after BS, at the Spaarne Gasthuis Hospital Hoofddorp, a dutch bariatric center. At our hospital, we have our own multidisciplinary team and 24/7 on call bariatric service. We perform 800 surgeries annually, with four bariatric surgeons, each with over 12 years of experience. Inclusion started in December 2020 and is ongoing. The entire episodes of all patients with abdominal pain after BS visiting the outpatient clinic or emergency department of the Spaarne Gasthuis are included. Figure 1 depicts examples of an episode. An episode starts during the first visit with abdominal pain within the study period and is completed when a patient did not visit the hospital because of abdominal pain for three consecutive months. After these 3 months, a new episode is initiated if a patient returns to the hospital with abdominal pain. All patients are followed throughout the entire episode of abdominal pain, which includes the first and all follow-up visits, peer consultations, diagnostic tests, readmissions, and reoperations for the pain. Enrolled patients receive regular treatment, and the study does not affect the patient’s visits, the choice of additional tests, follow-up, or treatment. At the end of each episode, it is determined whether the symptoms remained or whether the abdominal pain resolved since the last (follow-up) visit. Patients treated for abdominal pain in another hospital at the same time or with a BS procedure other than (revision) laparoscopic Roux-en-Y gastric bypass, laparoscopic sleeve gastrectomy, or laparoscopic one anastomosis gastric bypass are excluded from the study. The study protocol was approved by the Local Ethics Committee. The principles of the Declaration of Helsinki are followed.

The current substudy included patients who visited the hospital for abdominal pain between December 2020 and December 2021. Only the first episode of each patient with abdominal pain within this study period was included. In conclusion, in this study, each patient represents one episode. Ongoing episodes after November 1st, 2022, were excluded.

### Presumed Diagnoses, Definitive Diagnoses, and Unexplained Complaints

At the end of each visit and/or episode, it was assessed whether a patient met the criteria for one or more diagnoses. A distinction was made between presumed diagnoses and definitive diagnoses (Table 1). Two bariatric surgeons together with two researchers have jointly established the criteria for the most common diagnoses for abdominal pain, which was based on the golden standard. For example, (1) an internal herniation was called a presumed diagnosis if it was present on an abdominal CT-scan and a definitive diagnosis if it was confirmed during surgery and (2) an anastomotic ulcer was called a presumed diagnosis if symptoms improved during follow-up after treatment initiation and a definitive diagnosis after confirmation with a gastroscopy. When the criteria for a presumed diagnosis or definitive diagnosis were not met at the end of the episode, the complaints were classified as unexplained complaints. Because the official criteria for IBS and constipation, such as the ROME criteria, could not be derived from the clinical data, these diagnoses were only classified as a presumed diagnosis.

**Table 1:** Presumed and definitive diagnosis

	Presumed diagnosis (PreD)	Definitive diagnosis (DefD)
Abdominal wall herniation*	Ultrasound or computed tomography	Surgery
ACNES	Complaints consistent with diagnosis and a positive Pinchtest or Carnett sign	Lidocaine injection or surgery with a positive effect on the complaints
Appendicitis	Physical examination and blood test results	Ultrasound or computed tomography or surgery
Cholecystitis	Physical examination and blood test results	Ultrasound or computed tomography or surgery
Cholecystolithiasis	Ultrasound or computed tomography	Laparoscopic cholecystectomy or cholecystolithotomy or positive ERCP
Constipation	Complaints consistent with diagnosis and result of treatment with laxatives	-
Diverticulitis	Physical examination and blood test results	Ultrasound or computed tomography or surgery
IBS	Suspicion of IBS by the surgeon or gastroenterologist	-
Small bowel obstruction	Computed tomography	Surgery
Internal herniation	Computed tomography	Surgery
Leakage/perforation**	Computed tomography	Surgery
Nephrolithiasis	Complaints consistent with diagnosis and positive urine sediment with hemoglobin	Computed tomography
Pancreatitis	-	Blood test results
Stenosis GJ	Complaints consistent with diagnosis	Gastroscopy or barium swallow X-ray or computed tomography or surgery
Stenosis JJ	Complaints consistent with diagnosis	Computed tomography surgery
Ulcer	Complaints consistent with diagnosis and improvement of complaints with treatment (proton pump inhibitors and/or sucralfate)	Gastroscopy

*ACNES*, anterior cutaneous nerve entrapment syndrome; *ERCP*, endoscopic retrograde cholangio- and pancreatiography; *GJ*, gastrojejunostomy; *IBS*, irritable bowel disease; *JJ*, jejunojejunostomy

*Includes all sorts of abdominal wall herniations, for instance, umbilical hernia and cicatrical hernia

**Includes leakage after bariatric surgery but also after reoperations, for instance, bile leakage after laparoscopic cholecystectomie or perforation at the gastrojejunostomy because of an ulcer

### Outcomes

Primary outcome measures are the number of presumed diagnoses, definitive diagnoses, and unexplained complaints for abdominal pain in patients after BS during the first visit and at the end of an episode. Secondary outcomes are (1) the number of follow-up visits and their yield in the diagnostic process, (2) the number and duration of readmissions and number of reoperations, (3) the time it takes to establish a presumed diagnosis or definitive diagnosis, (4) the timeline between BS and the various diagnoses of abdominal pain, and finally, (5) whether complaints have resolved at the end of an episode.

### Data Collection

Baseline characteristics were collected from electronic patient records. Emergency department visits, readmission, and reoperations for abdominal pain after BS, before the start of the study period, were recorded. Before the start of the study, standardized reporting by the radiologist or gastroenterologist was implemented for abdominal CT-scans, ultrasounds, gastroscopies, barium swallow X-rays, and abdominal X-rays. Bariatric surgeons also completed a standardized surgery report in case of a reoperation. All data was stored in an electronic case record form in a web-based data capture-system (Castor EDC). The database was anonymized by a study number. Three researchers (NO, MV, and SV) having exclusive access to the database gathered the data and are able to re-identify patients based on their study number.

### Statistical Analysis

Data were reported as mean and standard deviation (SD) for normally distributed variables and as median and interquartile range (IQR) for variables with a skewed distribution. Continuous variables between groups were compared using the independent sample *t*-test, the non-parametric Mann-Whitney *U* test, or Kruskal Wallis test, depending on their distribution. The chi-square test was used for categorical variables. Logistic regression analysis was used to evaluate associations between patient characteristics and diagnoses. Variables with a *p* value of 0.10 or less in the univariable analysis were selected for the multivariable model. Stepwise backward elimination was used to derive a final model: in each subsequent step, the least significant variable in the model was removed until all remaining variables had individual *p* values smaller than 0.10. All *p* values are two-sided, and *p* values below 0.05 were considered statistically significant. Data were analyzed using Statistical Package for Social Sciences (SPSS) version 24 for windows (IBM Corporation, New York, USA). Tables and figures were made using Microsoft and SPSS.

## Results

### Description of the Study Population

From December 2020 until December 2021, 448 patients after BS visited the hospital for abdominal pain. Seven patients were excluded: three patients with incomplete episodes, two patients who were simultaneously treated in another hospital, and two patients with unclear anatomy of the gastric bypass. Baseline characteristics of the remaining 441 patients are presented in Table 2. The BS was performed in our hospital in 363 (87.7%) patients. Baseline characteristics of patients with their first visit at the outpatient clinic (204 (46.2%)) or emergency department (237 (53.7%)) were compared. A significant and clinically relevant difference was found only for the time after surgery in months, with 44.0 (IQR 16.5–73.0) and 26.0 (IQR 6.0–59.0) months for outpatient clinic and emergency department visits, respectively (*p* < 0.001).

**Table 2:** Patient characteristics

	Total patient group *n* = 441
Female sex—*n* (%)	401 (90.9)
Age (years)—median (IQR)	47.2 (37.1–55.0)
BMI before surgery (kg/m^2^)—median (IQR)	41.5 (38.6–45.0)
BMI at first presentation (kg/m^2^)—median (IQR)	28.3 (25.3–32.4)
% total weight loss—mean (SD)	31.4 (10.5)
Time after surgery in months—median (IQR)	37.0 (11.0–66.0)
Comorbidities—*n* (%)	
Hypertension	83 (18.8)
Type 2 diabetes mellitus	31 (7.0)
Fibromyalgie	32 (7.3)
A history of psychiatric diseases	102 (23.1)
Previous abdominal surgery*	242 (54.9)
Irritable bowel syndrome	31 (7.0)
Type of surgery—*n* (%)	
Roux-en-Y gastric bypass	380 (86.2)
Sleeve gastrectomy	20 (4.5)
One anastomosis gastric bypass	6 (1.4)
Revision bariatric surgery**	35 (7.9)
ED visits prior to the start of the study—*n* (%)	
0	321 (72.8)
1	73 (16.6)
2	28 (6.3)
≥ 3	19 (4.3)
Readmissions prior to the start of the study—*n* (%)	
0	306 (69.4)
1	88 (20.0)
2	26 (5.9)
≥ 3	21 (4.7)
Reoperations prior to the start of the study—*n* (%)	
0	322 (73.0)
1	84 (19.0)
2	18 (4.1)
≥ 3	17 (3.8)

*BMI*, body mass index in kg/m2; *ED*, emergency department; *IQR*, interquartile range

*Other than bariatric surgery

**All patients who previously had any form of bariatric surgery, converted to a laparoscopic Roux-en-Y gastric bypass, laparoscopic sleeve gastrectomy, or laparoscopic one anastomosis gastric bypass

### Presumed Diagnoses, Definitive Diagnoses, and Unexplained Complaints

At the end of the episode, 88 (20.0%) patients had a presumed diagnosis, 183 (41.5%) patients a definitive diagnosis, and 170 (38.5%) patients had unexplained complaints. Twenty-six patients had more than one diagnosis at the end of the episode. In 12 patients, the second or third diagnosis was a complication of the first diagnosis, for instance, bile leakage after cholecystectomy. The remaining patients had two or three unrelated diagnoses during their episode.

**Fig. 2:**
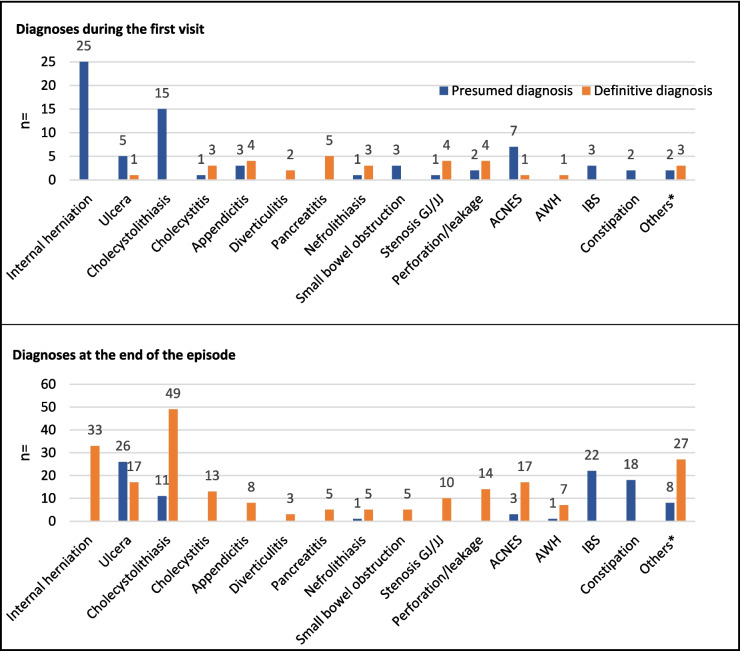
Presumed and definitive diagnoses. ACNES, anterior cutaneous nerve entrapment syndrome; AWH, abdominal wall herniation; OC, outpatient clinic; ED, emergency department; GJ, gastrojejunostomy; IBS, irritable bowel syndrome; JJ, jejunojejunostomy. **Omental infarction, reflux, intestinal torsion, diaphragmatic hernia, invagination, bleeding, cholangitis, ovarian torsion, abdominal wound dehiscence, urinetractinfection, gastritis, bezoar, mesenterial infiltration, vena porta trombosis, abdominal carcinoma, colitis, adhesions, lactose intollerance, herpes zoster, and COVID

Internal herniation and cholecystolithiasis were the most common presumed diagnoses during first visit (Fig. 2). The most common diagnoses at the end of follow-up were cholecystolithiasis in 60 (13.6%), ulcers in 43 (9.8%), and internal herniation in 33 (7.5%) patients.

### Follow-Up, Readmission, and Reoperation

**Fig. 3:**
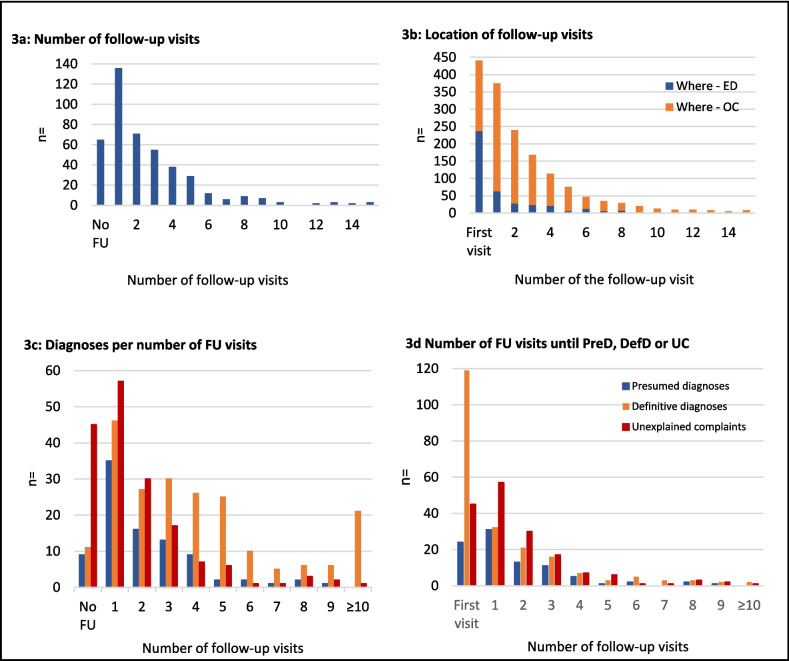
Number of follow-up visits and diagnoses per follow-up visit. FU, follow-up; DefD, definitive diagnosis; ED, emergency department; OC, outpatient clinic; PreD, presumed diagnosis; UC, unexplained complaints. **a** The number of follow-up visits during the entire episode per patient. Each patient is shown only once in this figure. **b** Figure for each follow-up visit whether this took place at the OC or at the ED. Every follow-up moment of all individual patients is shown, and therefore, patients can be presented multiple times in this figure. **c** Figure whether patients with a certain amount of follow-up visits had a presumed or definitive diagnoses or unexplained complaints at the end of an episode. **d** Figure on the basis of which follow-up visit in the episode a presumed or definitive diagnoses is made

A total of 136 patients (30.8%) had only one follow-up visit, whereas the majority had two to five follow-up visits in descending order (Fig. 3a). Unlike the first visit, most follow-up visits took place at the outpatient clinic (Fig. 3b). In addition, 77 (17.5%) patients had 1–3 peer consultations: gastroenterology (49.5%), general internal medicine (20.0%), pain management team (12.6%), urology (10.5%), gynecology (5.3%), and plastic surgery (2.1%). There were 260 readmissions across 212 patients, ranging from 1 to 4 per patient. Of these, 77 (29.6%) readmissions were for elective surgery. Readmissions lasted for 1 day in 120 (46.2%) cases and 51 (19.6%) lasted four or more days. In total, 186 reoperations were performed in 164 patients, with 1–3 reoperations per patient. Diagnostic laparoscopy was the most performed surgery followed by laparoscopic cholecystectomy (89 [47.8%] and 57 [30.6%], respectively). Data concerning readmissions and reoperations is shown in Supplemental Table S1.

Figure 3c shows that the number of presumed diagnoses established in patients with a certain amount of follow-up visits decreased, whereas the number of definitive diagnoses almost remained the same in patients with 2–5 follow-up visits. The percentage of patients with unexplained complaints decreased over the number of follow-up visits, whereas the proportion of definitive diagnoses increased. Despite the fact that patients often had more than 3 follow-up visits, Fig. 3d shows that a diagnosis was frequently made within the first visit or first three follow-up visits.

### Time Until Presumed Diagnoses, Definitive Diagnoses, and Unexplained Complaints

**Fig. 4:**
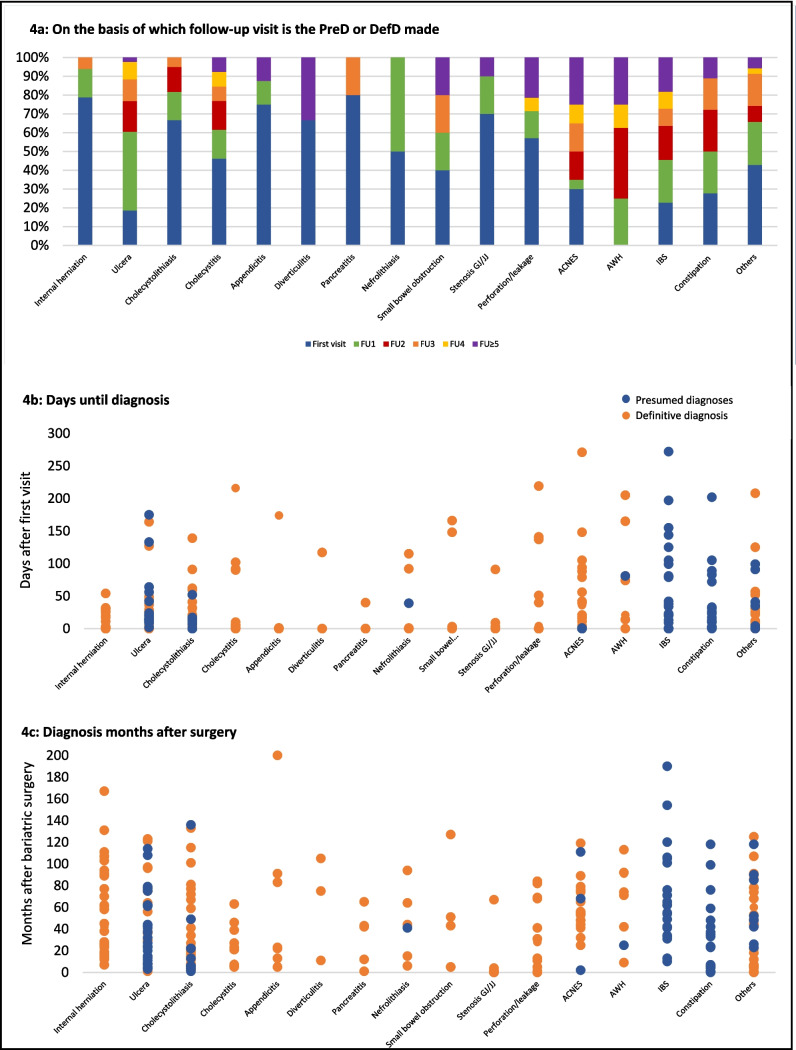
Diagnosis in time after first visit and after the bariatric surgery. ACNES, anterior cutaneous nerve entrapment syndrome; AWH, abdominal wall herniation; OC, outpatient clinic; DefD, definitive diagnosis; ED, emergency department; FU, follow-up; GJ, gastrojejunostomy; IBS, irritable bowel syndrome; JJ, jejunojejunostomy; PreD, presumed diagnosis. **a** The number of follow-up visits until a certain PreD or DefD is made is shown. **b** The days until a certain PreD or DefD is made. **c** The months after surgery in which certain diagnosis are made

Median (IQR) time to a presumed diagnosis, definitive diagnosis, or unexplained complaints from the start of the episode was 16.0 (3.8–44.5), 2.0 (0.0–31.5), and 13.5 (1.0–53.8) days, respectively (*p* < 0.001). Internal herniation, cholecystolithiasis, and appendicitis were usually diagnosed during the first visit or first follow-up visit. Anterior cutaneous nerve entrapment syndrome (ACNES), abdominal wall herniation, IBS, and constipation were usually diagnosed later on in the episode (Fig. 4a). These findings are consistent with Fig. 4b. Figure 4c demonstrates that there were no notable differences for specific diagnoses in time after BS. This was also not the case for the time after BS and a presumed diagnosis, definitive diagnosis, or unexplained complaints (*p* = 0.929).

### Association Between Patient Characteristics and Diagnoses

The multivariable model on the association between patient characteristics and diagnosis (presumed diagnoses/definitive diagnoses or unexplained complaints) revealed that patients with IBS more often had unexplained complaints at the end of the episode compared to patients without IBS (OR 95%CI: 4.46 [1.46–13.65], *p* = 0.009. The association between presence of a history of psychiatric diseases and unexplained complaints did not reach statistical significance (OR 95%CI: 1.63 [0.98–2.73]), *p* = 0.062). Patients with IBS had a significant higher probability of unexplained complaints when compared to patients with a definitive diagnosis (OR 95%CI: 4.27 [1.39–13.15], *p* = 0.011). A lower BMI at first presentation, IBS, and previous abdominal surgery were associated with a presumed diagnosis if compared to patients with a definitive diagnosis (OR 95%CI: 1.07 [1.01–1.13], *p* = 0.013; 6.74 [1.77–25.70], *p* = 0.005; and 1.45 [1.08–1.96], *p* = 0.014, respectively).

### Persisting or Resolved Abdominal Pain

At the end of the episode, 71 patients (16.1%) still experienced abdominal pain of which 29 (40.1%) had unexplained complaints. In 96 (21.7%) patients, it was not clear whether complaints had been resolved due to lack of follow-up or wait-and-see policy. Patients with IBS were most likely to have persistent complaints and in 5 (8.3%) patients treated for cholecystolithiasis complaints persisted without an alternative diagnosis. Data is shown in Supplemental Tables S2 and S3.

## Discussion

This is the first prospective study on the diagnoses of abdominal pain after BS which included the entire follow-up. In this study, a diagnosis for abdominal pain was found in 61.5% of patients, whereas 38.5% had unexplained complaints at the end of the episode. Most common diagnoses were cholecystolithiasis, ulcer, internal herniation, IBS, ACNES, and constipation. Majority of patients had one to five follow-up visits, but the diagnosis was usually established during the first four visits. Definitive diagnoses were set significantly earlier than presumed diagnoses and unexplained complaints. We did not observe an association between specific diagnoses and time after initial BS. Patients with unexplained complaints more often had a history of IBS. At the end of the episode, abdominal pain persisted in over 15% of patients and resolved in 62.2%.

In a systematic review on emergency department visits and readmissions after BS, only one study reported emergency department visits beyond 90 days after surgery, with 31.1% emergency department visits 3 years after RYGB [6, 21]. The readmission rate was 23.9% with a follow-up of up to 4.2 years. Main reasons for emergency department visits and readmissions were abdominal pain [6, 22–24]. Additionally, studies showed that 19.5–22% of patients had surgery for abdominal pain after BS [11, 25]. These data support the fact that emergency department visits, readmissions, and reoperations for abdominal pain after BS are more rule than exception.

Internal herniation, cholecystolithiasis, and ulcers were the top differential diagnoses during the first visit and turned out to be the most common diagnoses at the end of the episode. These three diagnoses were established early in the diagnostic process. In contrast, IBS, ACNES, and constipation were often diagnosed later in the episode. This may be explained by unspecific symptoms and the need for physicians to rule out potentially life-threatening causes for abdominal pain first. A retrospective cohort study compared symptoms in patients with unexplained complaints to patients with a specific diagnosis. The kind of pain (burning, nagging, etc.) and stool pattern were the only significantly differences between groups [20]. This supports the fact that complaints are often non-specific and can fit several diagnoses.

In patients after BS, many diagnostic tests are performed in the diagnostic process of abdominal pain [11, 26]. Furthermore, a diagnostic laparoscopy is regularly performed as a diagnostic tool for unexplained complaints [13, 27]. However, if a definitive diagnosis was made later in the episode, it was generally a complication of the initial diagnosis or a completely unrelated second diagnosis. As a result, patients with consistent complaints after the first follow-up visit are more likely to have a non-acute cause, for instance, IBS, ACNES, and constipation. In addition, the abdominal pain disappeared spontaneously in one in five patients. Therefore, a wait-and-see policy rather than diagnostic tests might be a better option in patients without warning symptoms.

The current literature shows (chronic) abdominal complaints in 33.8–54.4% after BS of which 4.5–53% sought medical help for these complaints [7, 9, 28]. These studies show that a substantial number of patients with abdominal pain never seek medical help for their symptoms. This could explain why only 38 (8.6%) patients in the present study had chronic complaints without a diagnosis at the end of the episode. However, we expect that the actual percentage of chronic complaints is probably higher due to two reasons. First, it is not clear whether complaints had been resolved in 66 patients with unexplained complaints due to lack of follow-up or wait-and-see policy. Second, patients with abdominal pain during the annual follow-up visits with the internist at the outpatient clinic were only included when they were referred to the surgeon. Moreover, this substudy only included patient’s first episode within the study period, so further research on subsequent episodes will probably show that this percentage is even higher. The fact that a high percentage of patients in our study had an emergency department visit, readmission, or reoperation before this study does indicate chronic or recurrent complaints as well.

In the analyses of this study, we did not take into account whether patients had abdominal pain before BS. According to a study by Hogestol et al., 18.5% of patients with chronic abdominal pain also had complaints prior to surgery [9]. In another study, the percentage of patients with chronic abdominal complaints before BS was 11.9% [10].

Out of the scope of this article, but remarkable, is the fact that many patients experience all kinds of other gastrointestinal symptoms in addition to abdominal pain [8–10, 29, 30]. This could indicate a more systemic and metabolic cause for these symptoms such as pancreatic insufficiency or changes in the gut microbiome and needs more research [31, 32].

### Limitations of the Study

All previous visits, readmissions, reoperations, and diagnoses for abdominal pain before the start of the study are not included in the analyses. This makes the study less comparable to current retrospective cohort studies. On the other hand, this design results in a larger group of patients, and it better reflects daily practice in the outpatient clinic and emergency department. In patients with previous unexplained complaints, more acute causes were possible already ruled out during earlier episodes. Therefore, the diagnostic process could have focused more on another set of diagnoses than in patients with a first presentation for abdominal pain. Future research on sequential episodes in the OPERATE study will provide more information on the impact of these patients. This study was started in the COVID pandemic. This could have impacted the number of visits and the duration of the episodes. The numbers of several diagnoses were too small to evaluate in what time after surgery they occurred. Furthermore, although no associations were observed between type of operation and outcomes, Roux-en-Y gastric bypass is still the number one of BS performed in the Netherlands, and there are too little sleeve gastrectomies and one-anastomosis gastric bypass surgeries to compare with. This is important because current literature shows that the numbers of emergency department visits and readmissions for abdominal pain after sleeve gastrectomy are quite similar to those of the Roux-en-Y gastric bypass [33]. Lastly, socio-economic factors are not included in this study.

## Conclusion

Most common diagnoses for abdominal pain after BS were cholelithiasis, ulcers, internal herniations, IBS, ACNES, and constipation. However, over a third of abdominal pain remains unexplained, and at the end of the episode, over 15% of patients continues to have complaints. In the future, people with an acute cause for abdominal pain, such as an internal herniation, should be better distinguished from patients with transient or chronic complaints to prevent unnecessary visits, diagnostic tests, and reoperations. Therefore, there is a need for an algorithm to better distinguish, diagnose, and ultimately treat patient, one of the main goals of the OPERATE study.

### Supplementary Information

Below is the link to the electronic supplementary material.

Supplementary file1 (DOCX 21 KB)

Supplementary file2 (DOCX 22 KB)

Supplementary file3 (DOCX 24 KB)

## Data Availability

We do not present any data in a public repository.

## Abbreviations

ACNES: Anterior cutaneous nerve entrapment syndrome
AWH: Abdominal wall herniation
OC: Outpatient clinic
BS: Bariatric surgery
BMI: Body mass index
DefD: Definitive diagnosis
ED: Emergency department
FU: Follow-up
GJ: Gastrojejunostomy
IFSO: International Federation for the Surgery of Obesity and Metabolic Disorders
IQR: Interquartile range
IBS: Irritable bowel syndrome
JJ: Jejunojejunostomy
LSG: Laparoscopic sleeve gastrectomy
LOAGB: laparoscopic one anastomosis gastric bypass
LRYGB: laparoscopic Roux-en-Y gastric bypass
NA: Not applicable
%TWL: Percentage total weight loss
PreD: Presumed diagnosis
UC: Unexplained complaints
