# Supplementary material for: A Prospective Study on the Diagnoses for Abdominal Pain After Bariatric Surgery: The OPERATE Study
Source: Obes Surg. 2023 Aug 11;33(10):3017–27. doi: 10.1007/s11695-023-06756-3 (PMC10514148; doi:10.1007/s11695-023-06756-3)
Supplement: Supplementary file 1 — Supplementary file1 (DOCX 21 KB) [file 11695_2023_6756_MOESM1_ESM.docx]

***Supplemental table S1: Readmissions and reoperations***

|  | **n (%)** |
| --- | --- |
| **Number of readmissions** |  |
| 0 | 229 (51.9) |
| 1 | 175 (39.7) |
| 2 | 28 (6.3) |
| 3 | 7 (1.6) |
| 4 | 2 (0.5) |
| Total | 260 |
|  |  |
| **Readmission for elective surgery** | 77 (29.6) |
|  |  |
| **Number of admitted days** |  |
| Same day discharge | 21 (8.1) |
| 1 | 120 (46.2) |
| 2 | 45 (17.3) |
| 3 | 23 (8.8) |
| >4 | 51 (19.6) |
|  |  |
| **Number of reoperations** |  |
| 0 | 277 (62.8) |
| 1 | 147 (33.3) |
| 2 | 12 (2.7) |
| 3 | 5 (1.1) |
| Total | 186 |
|  |  |
| Diagnostic laparoscopy | 89 (47.8) |
| Laparoscopic cholecystectomy | 57 (30.6) |
| Other | 40 (21.5) |
