# Supplementary material for: A Prospective Study on the Diagnoses for Abdominal Pain After Bariatric Surgery: The OPERATE Study
Source: Obes Surg. 2023 Aug 11;33(10):3017–27. doi: 10.1007/s11695-023-06756-3 (PMC10514148; doi:10.1007/s11695-023-06756-3)
Supplement: Supplementary file 2 — Supplementary file2 (DOCX 22 KB) [file 11695_2023_6756_MOESM2_ESM.docx]

|  | Total  n (%) | PreD  n (%) | DefD  n (%) | UC  n (%) |
| --- | --- | --- | --- | --- |
| ***Persistent*** |  |  |  |  |
| Abdominal complaints consistent with diagnosis | 33 (7.5) | 21 (23.9) | 12 (6.6) | 0 (0.0) |
| Chronic abdominal complaints (without diagnosis) | 38 (8.6) | 3 (3.4) | 6 (3.3) | 29 (17.1) |
| ***Resolved*** |  |  |  |  |
| Complaints are under control with continuous treatment | 42 (9.5) | 31 (35.2) | 9 (4.9) | 2 (1.2) |
| Complaints resolved after treatment of PreD or DefD | 144 (32.7) | 9 (10.2) | 135 (73.8) | 0 (0.0) |
| Complaints disappeared spontaneously | 88 (20.0) | 11 (12.5) | 4 (2.2) | 73 (42.9) |
| ***Unclear*** |  |  |  |  |
| Unclear whether there are still complaints due to lack of follow-up | 50 (11.3) | 4 (4.5) | 14 (7.7) | 32 (18.8) |
| Unclear if there are still complaints due to a wait-and-see policy and no presentation at the hospital afterwards | 46 (10.4) | 9 (10.2) | 3 (1.6) | 34 (20.0) |

***Supplemental table S2: Persisting or resolved abdominal pain at the end of follow-up.***

*DefD= definitive diagnosis, PreD= presumed diagnosis, UC= unexplained complaints*
