# Supplementary material for: A Prospective Study on the Diagnoses for Abdominal Pain After Bariatric Surgery: The OPERATE Study
Source: Obes Surg. 2023 Aug 11;33(10):3017–27. doi: 10.1007/s11695-023-06756-3 (PMC10514148; doi:10.1007/s11695-023-06756-3)
Supplement: Supplementary file 3 — Supplementary file3 (DOCX 24 KB) [file 11695_2023_6756_MOESM3_ESM.docx]

***Supplemental table S3: Persisting or resolved complaints at the end of follow-up***

|  | **Abdominal complaints consistent with diagnosis** | **Chronic abdominal complaints (without diagnosis)** | **Complaints are under control with continuous treatment** | **Complaints resolved after treatment of PD or DD** | **Complaints disappeared spontaneously** | **Unclear whether there are still complaints due to lack of follow-up** | **Unclear if there are still complaints due to a wait-and-see policy and no presentation at the hospital afterwards** |
| --- | --- | --- | --- | --- | --- | --- | --- |
| **Internal herniation n (%)** | 0 (0.0) | 0 (0.0) | 0 (0.0) | 31 (93.9) | 0 (0.0) | 2 (6.1) | 0 (0.0) |
| **Ulcera n (%)** | 2 (4.7) | 1 (2.3) | 25 (58.1) | 12 (27.9) | 0 (0.0) | 3 (7.0) | 0 (0.0) |
| **Cholecystolithiasis n (%)** | 2 (3.3) | 5 (8.3) | 0 (0.0) | 41 (68.3) | 7 (11.7) | 4 (6.7) | 1 (1.7) |
| **Cholecystitis n (%)** | 0 (0.0) | 1 (7.7) | 0 (0.0) | 11 (84.6) | 0 (0.0) | 0 (0.0) | 1 (7.7) |
| **Appendicitis n (%)** | 0 (0.0) | 0 (0.0) | 0 (0.0) | 8 (100%) | 0 (0.0) | 0 (0.0) | 0 (0.0) |
| **Diverticulitis n (%)** | 1 (33.3) | 0 (0.0) | 0 (0.0) | 0 (0.0) | 0 (0.0) | 1 (33.3) | 1 (33.3) |
| **Pancreatitis n (%)** | 1 (20.0) | 0 (0.0) | 0 (0.0) | 3 (60.0) | 0 (0.0) | 1 (20.0) | 0 (0.0) |
| **Nefrolithiasis n (%)** | 3 (50.0) | 0 (0.0) | 0 (0.0) | 2 (33.3) | 1 (16.7) | 0 (0.0) | 0 (0.0) |
| **Small bowel obstruction n(%)** | 0 (0.0) | 0 (0.0) | 1 (20.0) | 4 (80.0) | 0 (0.0) | 0 (0.0) | 0 (0.0) |
| **Stenosis GJ/JJ n (%)** | 0 (0.0) | 0 (0.0) | 0 (0.0) | 7 (70.0) | 2 (20.0) | 1 (10.0) | 0 (0.0) |
| **Perforation/leakage n (%)** | 0 (0.0) | 0 (0.0) | 0 (0.0) | 13 (92.8) | 0 (0.0) | 0 (0.0) | 1 (7.1) |
| **ACNES n (%)** | 4 (20.0) | 0 (0.0) | 2 (10.0) | 11 (55.0) | 1 (5.0) | 1 (5.0) | 1 (5.0) |
| **AWH n (%)** | 1 (12.5) | 0 (0.0) | 0 (0.0) | 6 (75.0) | 0 (0.0) | 1 (12.5) | 0 (0.0) |
| **IBS n (%)** | 10 (45.5) | 1 (4.5) | 4 (18.2) | 0 (0.0) | 2 (9.1) | 1 (4.5) | 4 (18.2) |
| **Constipation n (%)** | 2 (11.1) | 0 (0.0) | 6 (33.3) | 5 (27.8) | 1 (5.6) | 2 (11.1) | 2 (11.1) |
| **Others n (%)** | 7 (20.0) | 2 (5.7) | 4 (11.4) | 18 (51.5) | 1 (2.9) | 1 (2.9) | 2 (5.7) |

*ACNES= anterior cutaneous nerve entrapment syndrome, AWH= abdominal wall herniation, OC= outpatient clinic, DefD= Definitive diagnosis, ED= emergency department, FU= follow-up, GJ= gastrojejunostomy, IBS= irritable bowel syndrome,
JJ= jejunojejunostomy, PreD= presumed diagnosis*
